# Supplementary material for: A novel triptolide analog downregulates NF-κB and induces mitochondrial apoptosis pathways in human pancreatic cancer
Source: eLife. 2023 Oct 25;12:e85862. doi: 10.7554/eLife.85862 (PMC10861173; doi:10.7554/eLife.85862)
Supplement: Figure 1—source data 1. — Acute maximum tolerated dose (MTD) studies, toxicity and toxicokinetic studies on rats and beagle dogs. [file elife-85862-fig1-data1.pptx]

## Slide 1
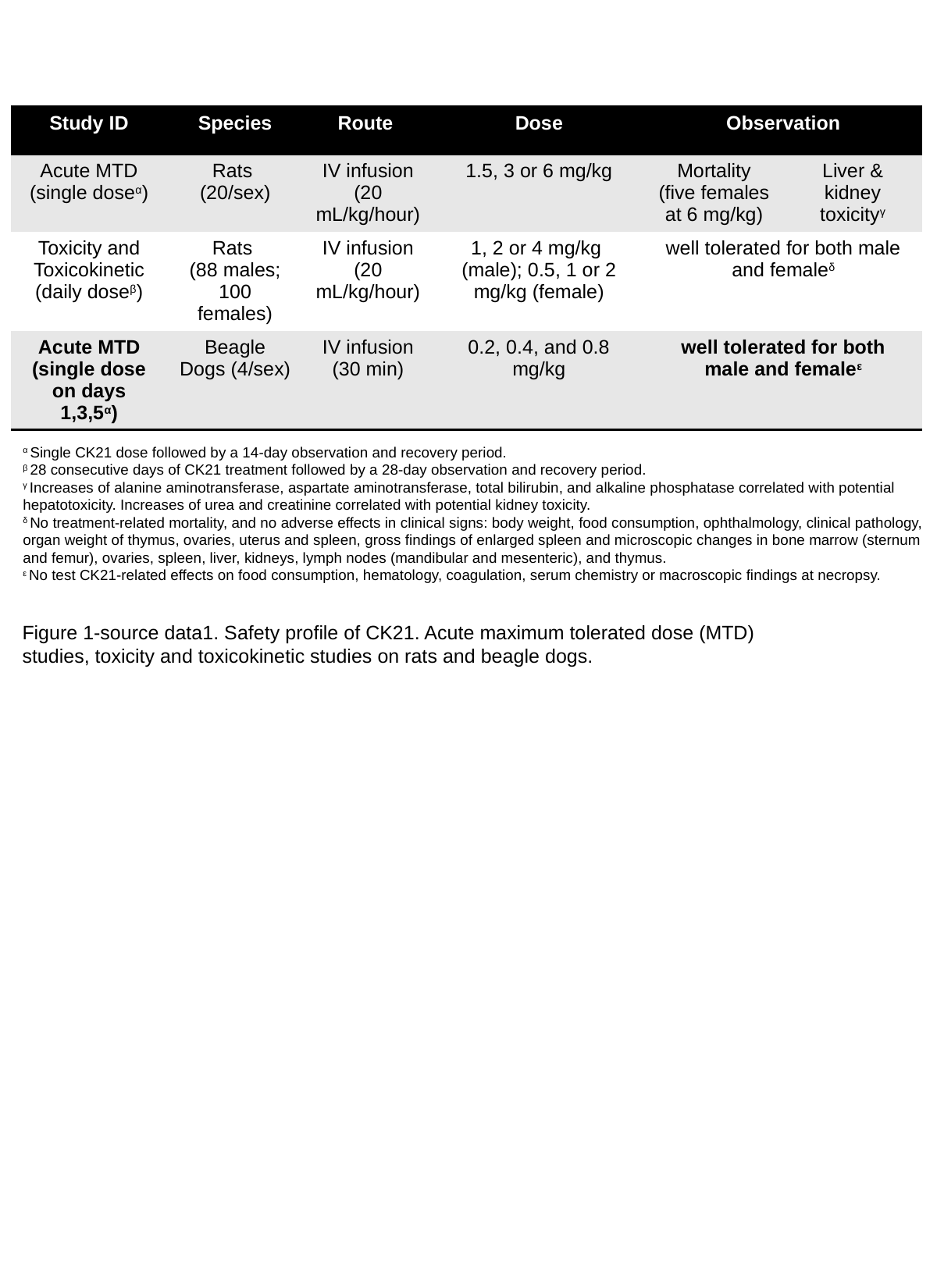

| Study ID | Species | Route | Dose | Observation | |
| --- | --- | --- | --- | --- | --- |
| Acute MTD (single doseα) | Rats (20/sex) | IV infusion (20 mL/kg/hour) | 1.5, 3 or 6 mg/kg | Mortality (five females at 6 mg/kg) | Liver & kidney toxicityγ |
| Toxicity and Toxicokinetic (daily doseβ) | Rats (88 males; 100 females) | IV infusion (20 mL/kg/hour) | 1, 2 or 4 mg/kg (male); 0.5, 1 or 2 mg/kg (female) | well tolerated for both male and femaleδ | |
| Acute MTD (single dose on days 1,3,5α) | Beagle Dogs (4/sex) | IV infusion (30 min) | 0.2, 0.4, and 0.8 mg/kg | well tolerated for both male and femaleε | |
α Single CK21 dose followed by a 14-day observation and recovery period.
β 28 consecutive days of CK21 treatment followed by a 28-day observation and recovery period.
γ Increases of alanine aminotransferase, aspartate aminotransferase, total bilirubin, and alkaline phosphatase correlated with potential hepatotoxicity. Increases of urea and creatinine correlated with potential kidney toxicity.
δ No treatment-related mortality, and no adverse effects in clinical signs: body weight, food consumption, ophthalmology, clinical pathology, organ weight of thymus, ovaries, uterus and spleen, gross findings of enlarged spleen and microscopic changes in bone marrow (sternum and femur), ovaries, spleen, liver, kidneys, lymph nodes (mandibular and mesenteric), and thymus.
ε No test CK21-related effects on food consumption, hematology, coagulation, serum chemistry or macroscopic findings at necropsy.
Figure 1-source data1. Safety profile of CK21. Acute maximum tolerated dose (MTD) studies, toxicity and toxicokinetic studies on rats and beagle dogs.
