# Supplementary material for: A novel triptolide analog downregulates NF-κB and induces mitochondrial apoptosis pathways in human pancreatic cancer
Source: eLife. 2023 Oct 25;12:e85862. doi: 10.7554/eLife.85862 (PMC10861173; doi:10.7554/eLife.85862)
Supplement: Figure 1—source data 2. [file elife-85862-fig1-data2.pptx]

## Slide 1
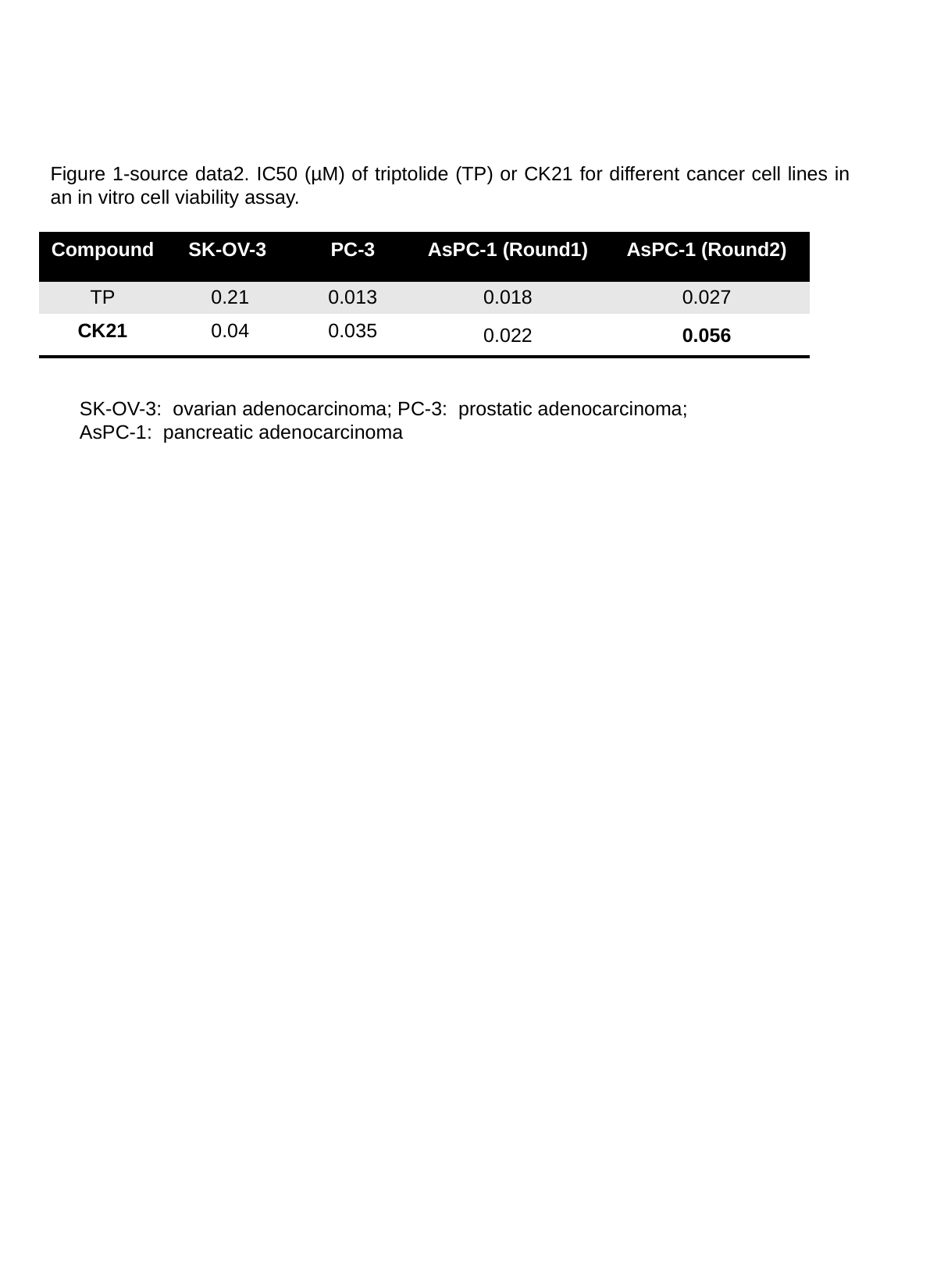

Figure 1-source data2. IC50 (µM) of triptolide (TP) or CK21 for different cancer cell lines in an in vitro cell viability assay.
| Compound | SK-OV-3 | PC-3 | AsPC-1 (Round1) | AsPC-1 (Round2) |
| --- | --- | --- | --- | --- |
| TP | 0.21 | 0.013 | 0.018 | 0.027 |
| CK21 | 0.04 | 0.035 | 0.022 | 0.056 |
SK-OV-3: ovarian adenocarcinoma; PC-3: prostatic adenocarcinoma;
AsPC-1: pancreatic adenocarcinoma
