# Supplementary material for: A novel triptolide analog downregulates NF-κB and induces mitochondrial apoptosis pathways in human pancreatic cancer
Source: eLife. 2023 Oct 25;12:e85862. doi: 10.7554/eLife.85862 (PMC10861173; doi:10.7554/eLife.85862)
Supplement: Figure 4—source data 1. — Details of organoids from Patient# 1, 2, 6, and 7 are provided in reference 39 (Romero-Calvo et al., 2019). [file elife-85862-fig4-data1.pptx]

## Slide 1
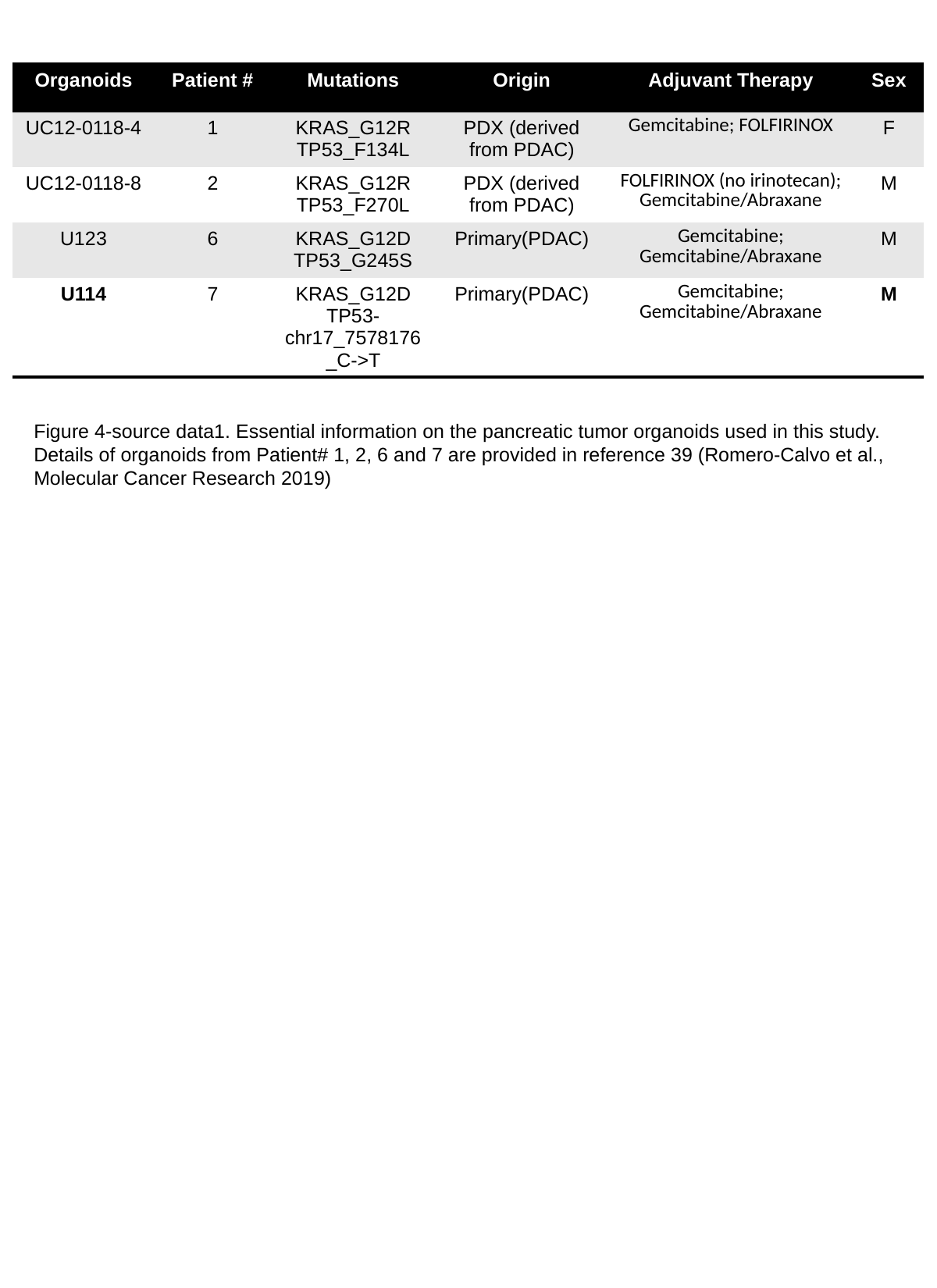

| Organoids | Patient # | Mutations | Origin | Adjuvant Therapy | Sex |
| --- | --- | --- | --- | --- | --- |
| UC12-0118-4 | 1 | KRAS\_G12R TP53\_F134L | PDX (derived from PDAC) | Gemcitabine; FOLFIRINOX | F |
| UC12-0118-8 | 2 | KRAS\_G12R TP53\_F270L | PDX (derived from PDAC) | FOLFIRINOX (no irinotecan); Gemcitabine/Abraxane | M |
| U123 | 6 | KRAS\_G12D TP53\_G245S | Primary(PDAC) | Gemcitabine; Gemcitabine/Abraxane | M |
| U114 | 7 | KRAS\_G12D TP53-chr17\_7578176\_C->T | Primary(PDAC) | Gemcitabine; Gemcitabine/Abraxane | M |
Figure 4-source data1. Essential information on the pancreatic tumor organoids used in this study. Details of organoids from Patient# 1, 2, 6 and 7 are provided in reference 39 (Romero-Calvo et al., Molecular Cancer Research 2019)
