# Supplementary material for: A novel triptolide analog downregulates NF-κB and induces mitochondrial apoptosis pathways in human pancreatic cancer
Source: eLife. 2023 Oct 25;12:e85862. doi: 10.7554/eLife.85862 (PMC10861173; doi:10.7554/eLife.85862)
Supplement: Figure 6—source data 1. [file elife-85862-fig6-data1.zip › Full unedited gel for Figure 6 h and i.pptx]

## Slide 1
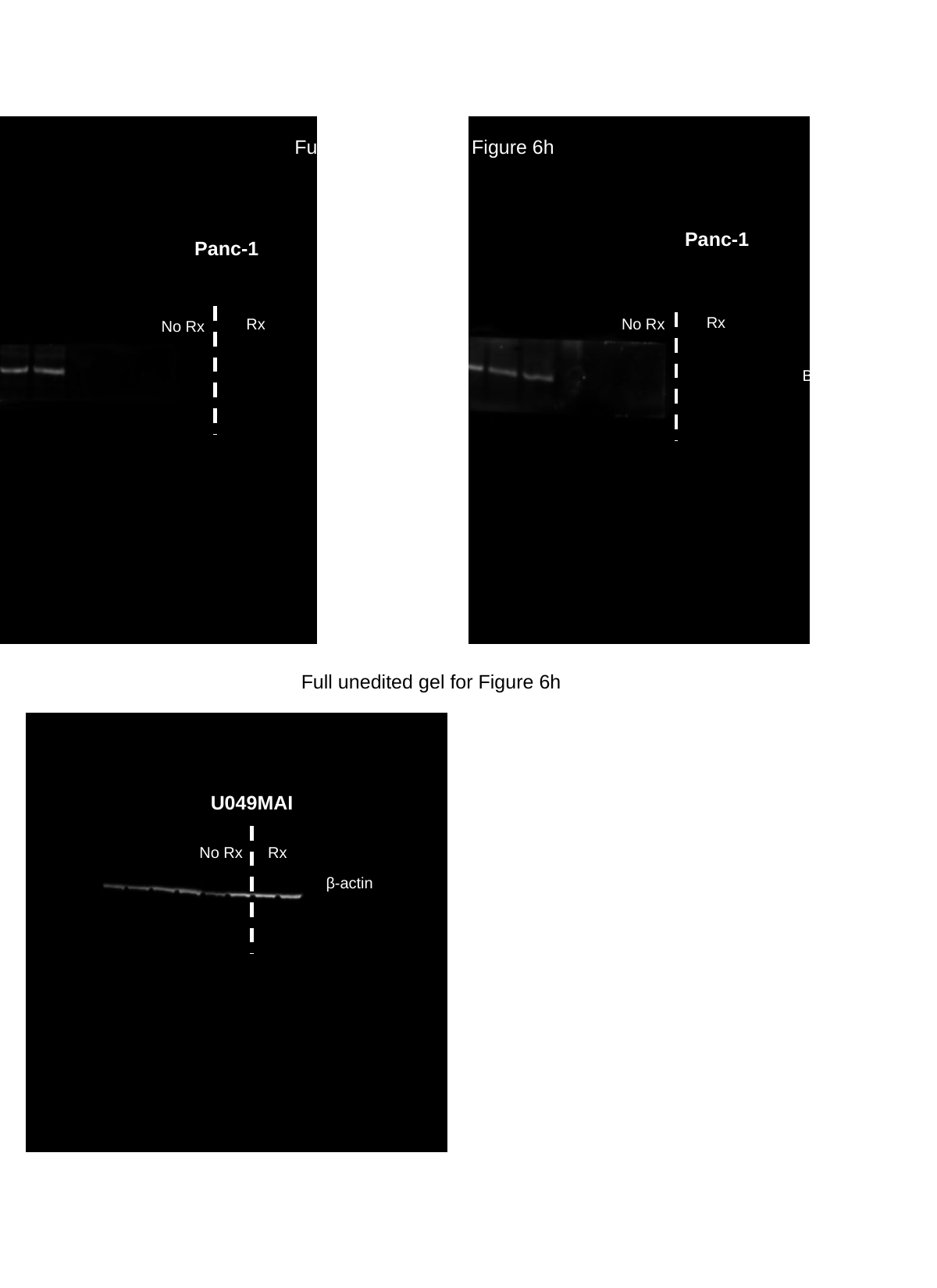

Full unedited gel for Figure 6h
Panc-1
Panc-1
Rx
No Rx
Rx
No Rx
β-actin
BCL2
Full unedited gel for Figure 6h
U049MAI
U049MAI
No Rx
Rx
No Rx
Rx
β-actin
BCL2

## Slide 2
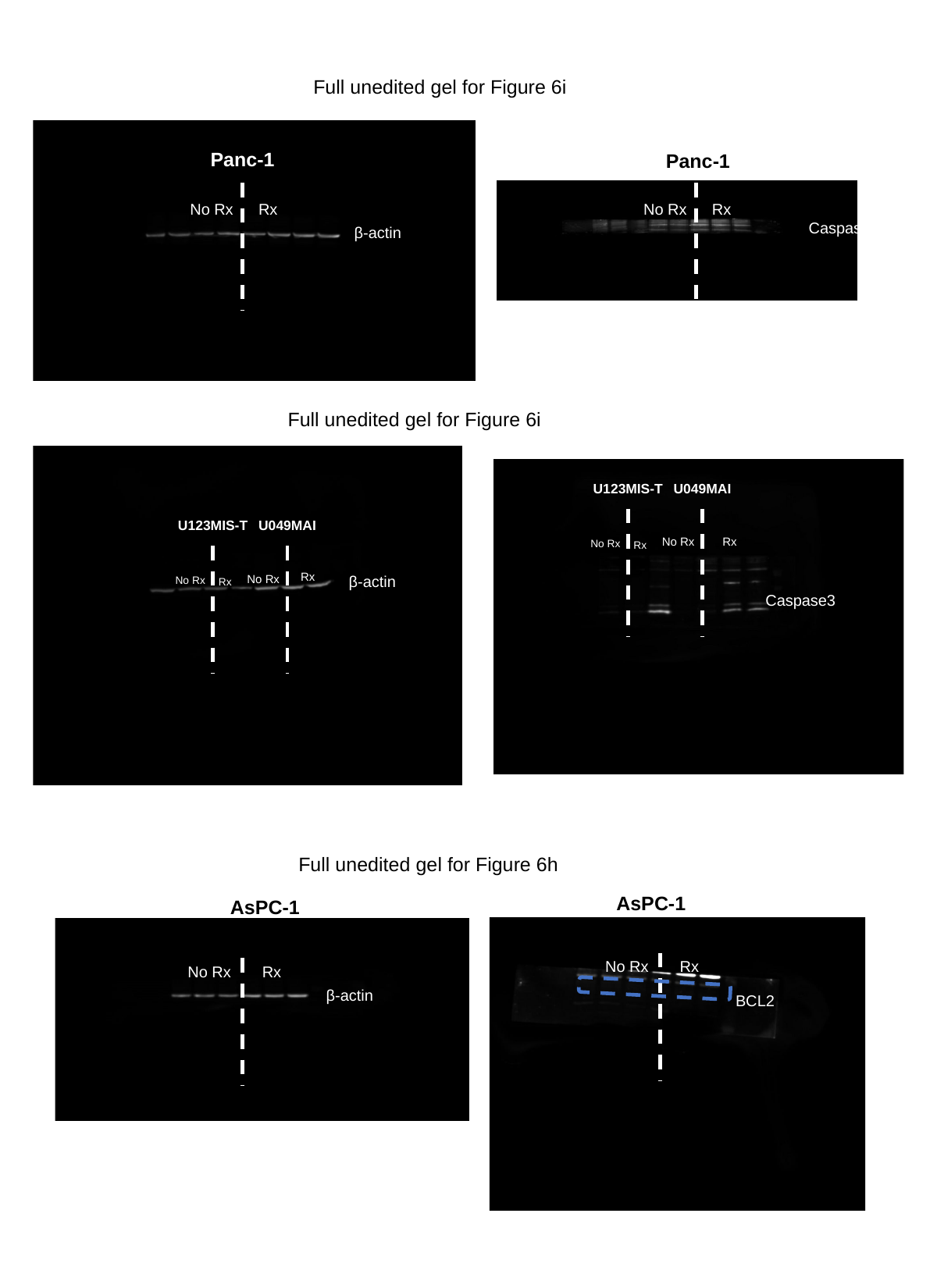

Full unedited gel for Figure 6i
Panc-1
Panc-1
No Rx
Rx
No Rx
Rx
Caspase3
β-actin
Full unedited gel for Figure 6i
U123MIS-T
U049MAI
U123MIS-T
U049MAI
No Rx
Rx
No Rx
Rx
Rx
No Rx
β-actin
No Rx
Rx
Caspase3
Full unedited gel for Figure 6h
AsPC-1
AsPC-1
Rx
No Rx
Rx
No Rx
BCL2
β-actin
BCL2
