# Supplementary figures and images for: A novel triptolide analog downregulates NF-κB and induces mitochondrial apoptosis pathways in human pancreatic cancer
Source: eLife. 2023 Oct 25;12:e85862. doi: 10.7554/eLife.85862 (PMC10861173; doi:10.7554/eLife.85862)

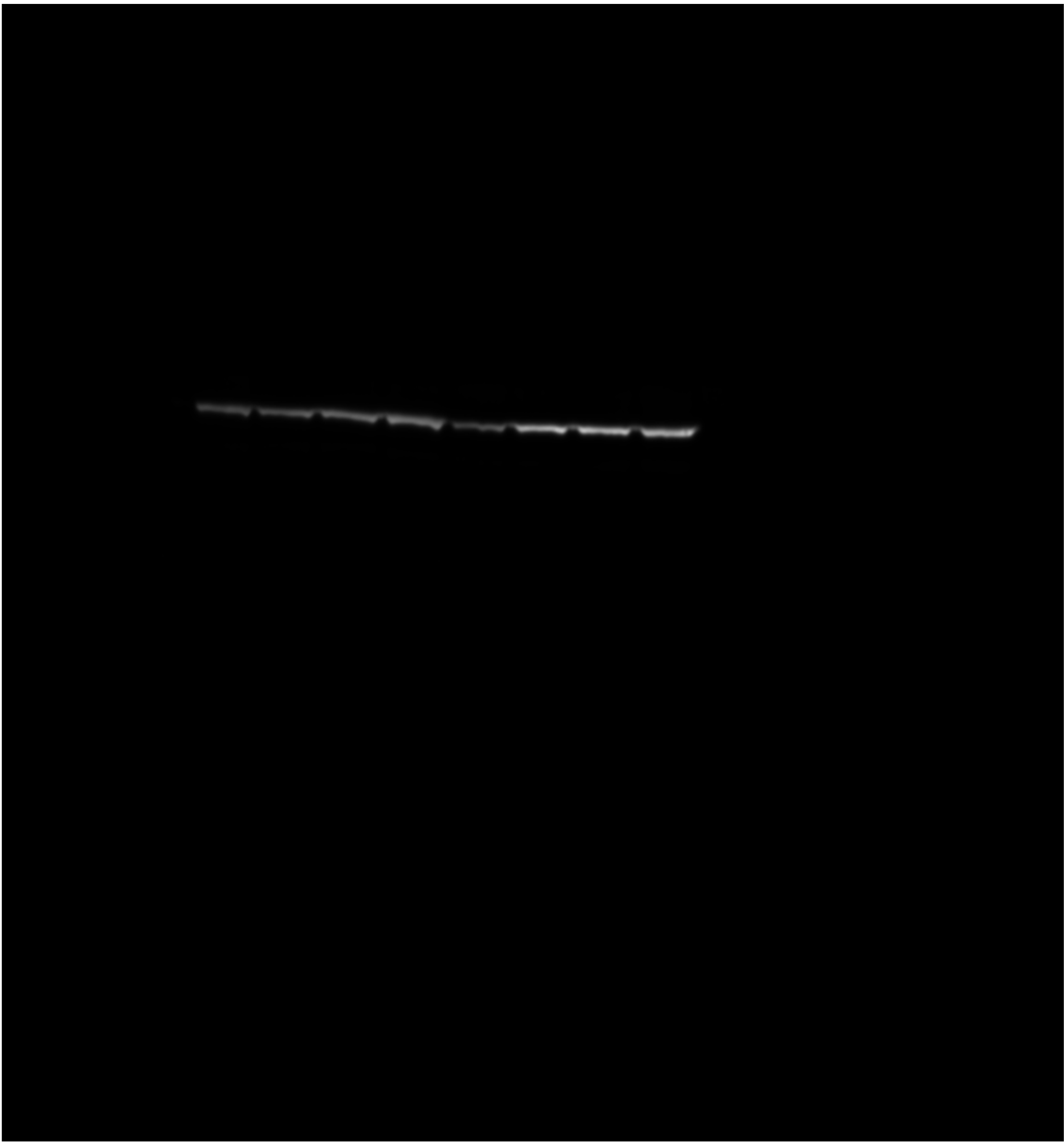

Supplement: Figure 6—source data 1. [file elife-85862-fig6-data1.zip › 6h/U049MAI-bactin.tif]

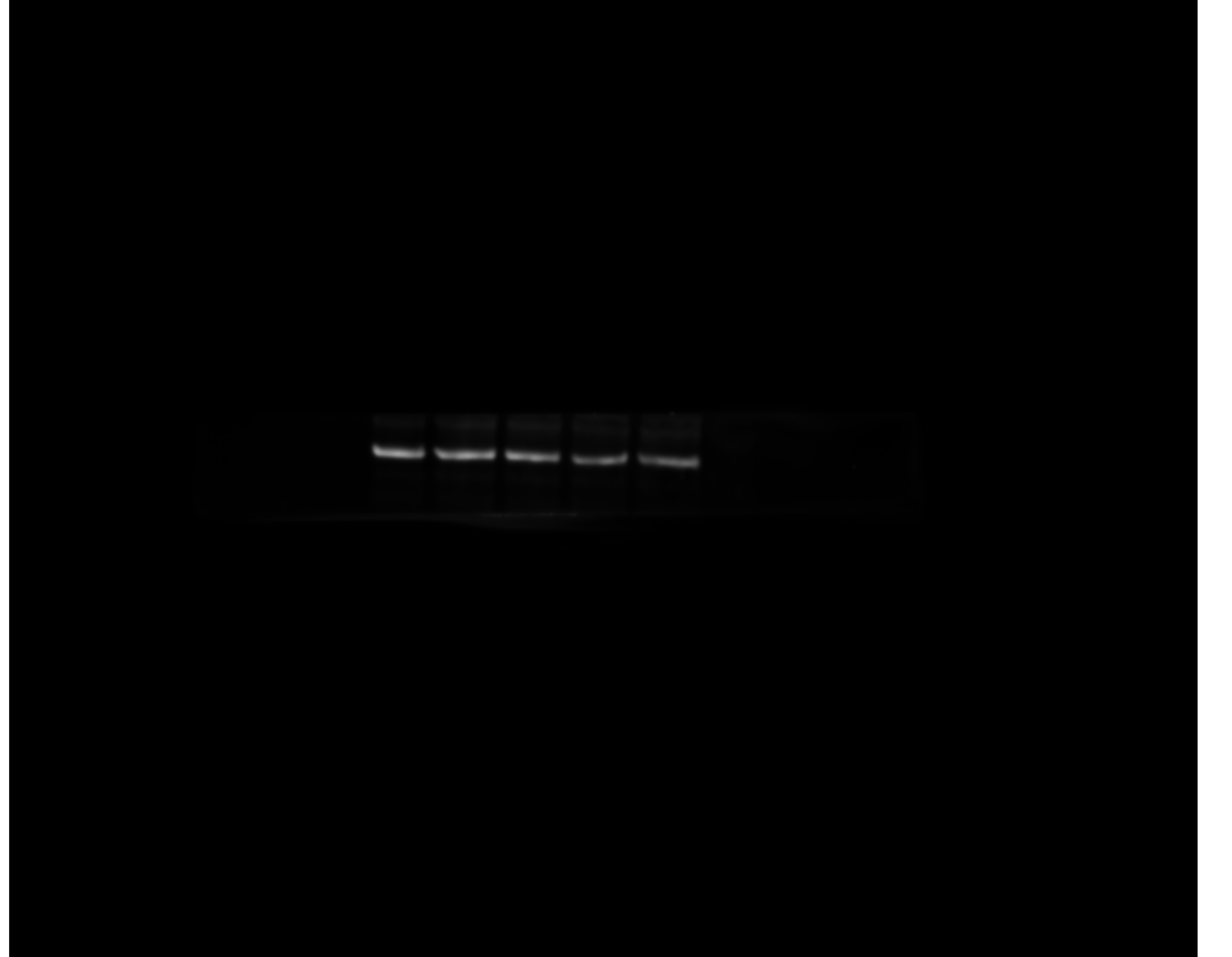

Supplement: Figure 6—source data 1. [file elife-85862-fig6-data1.zip › 6h/Panc-1-bactin.tif]

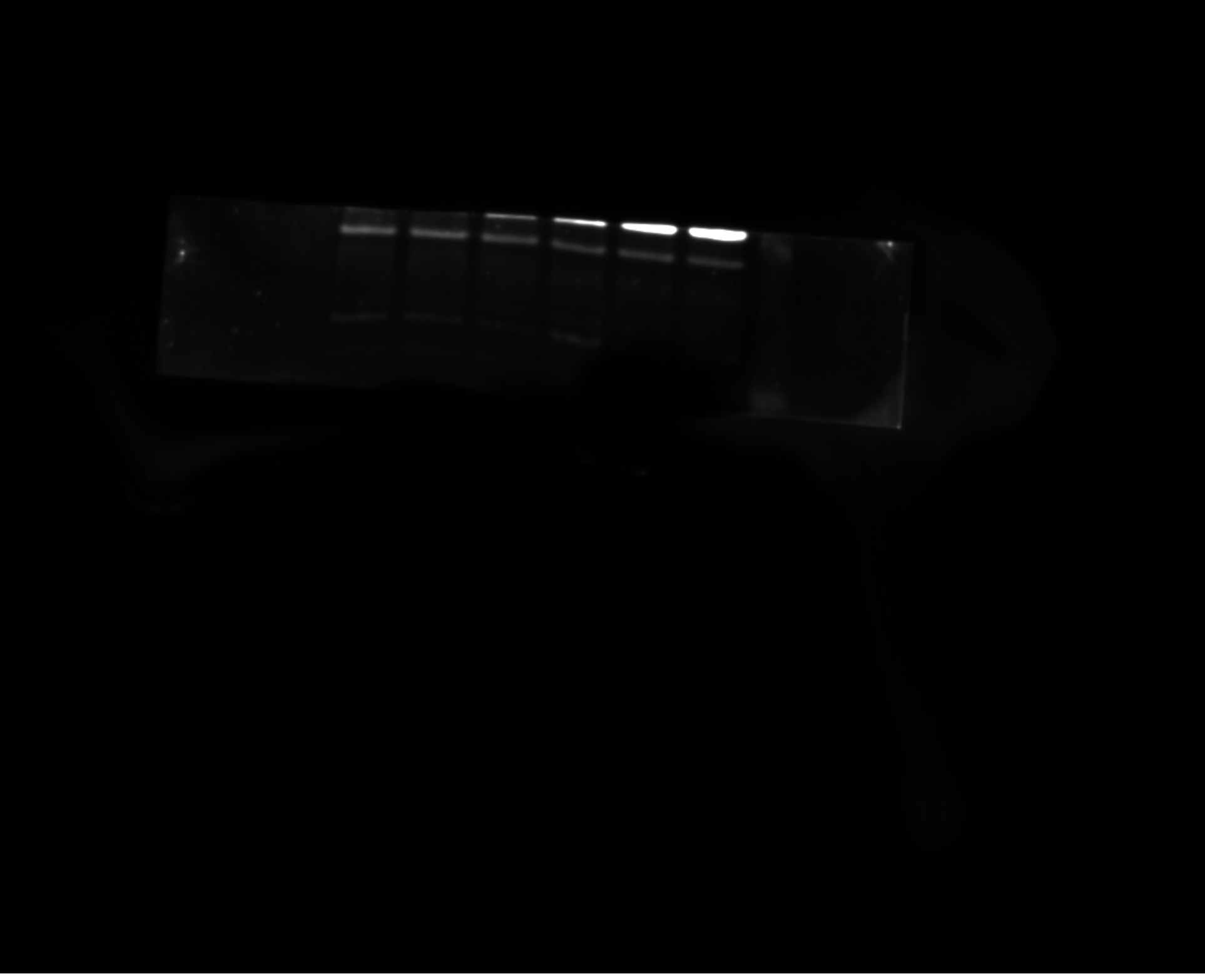

Supplement: Figure 6—source data 1. [file elife-85862-fig6-data1.zip › 6h/AsPC-1-BCL2.tif]

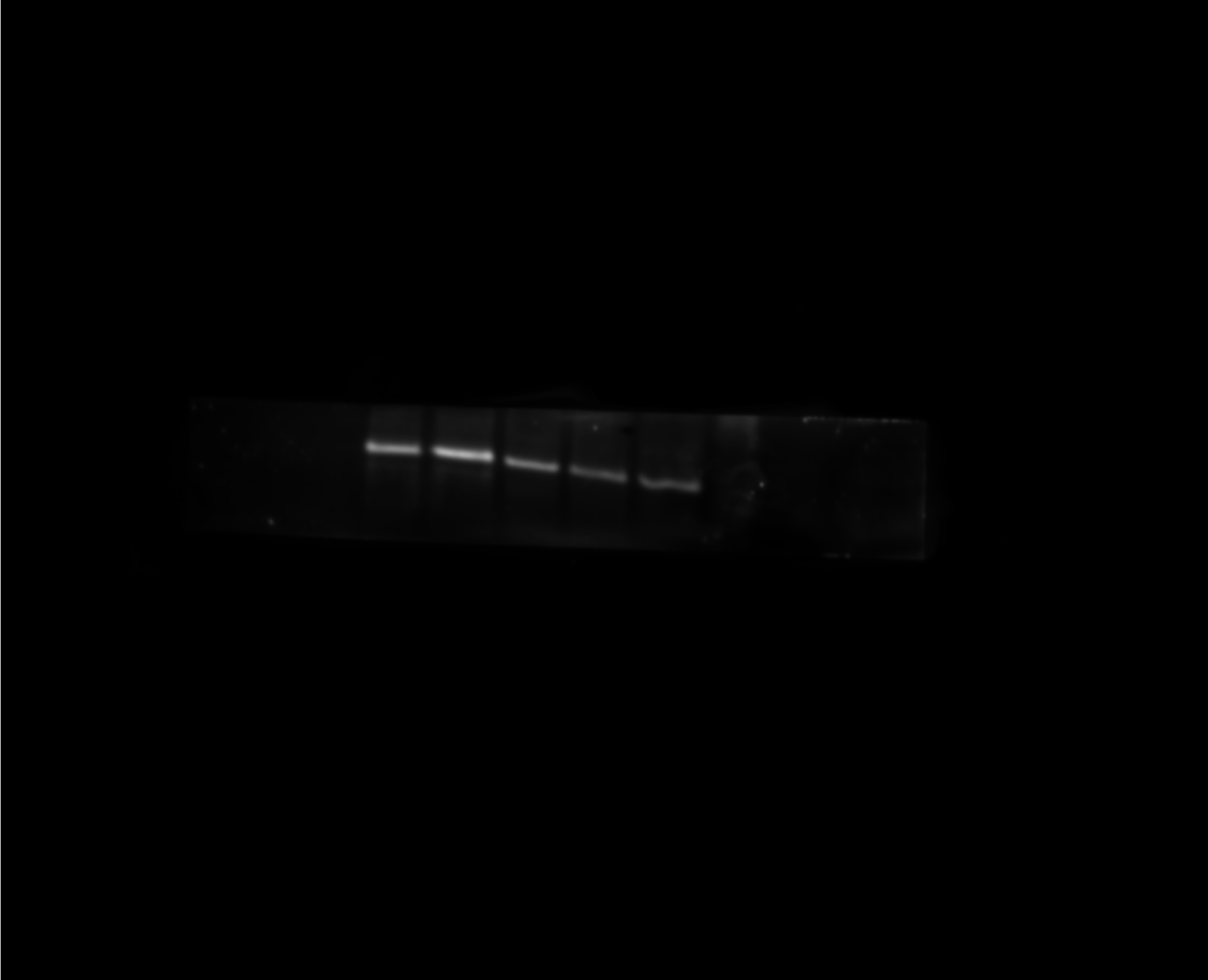

Supplement: Figure 6—source data 1. [file elife-85862-fig6-data1.zip › 6h/Panc-1-BCL2.tif]

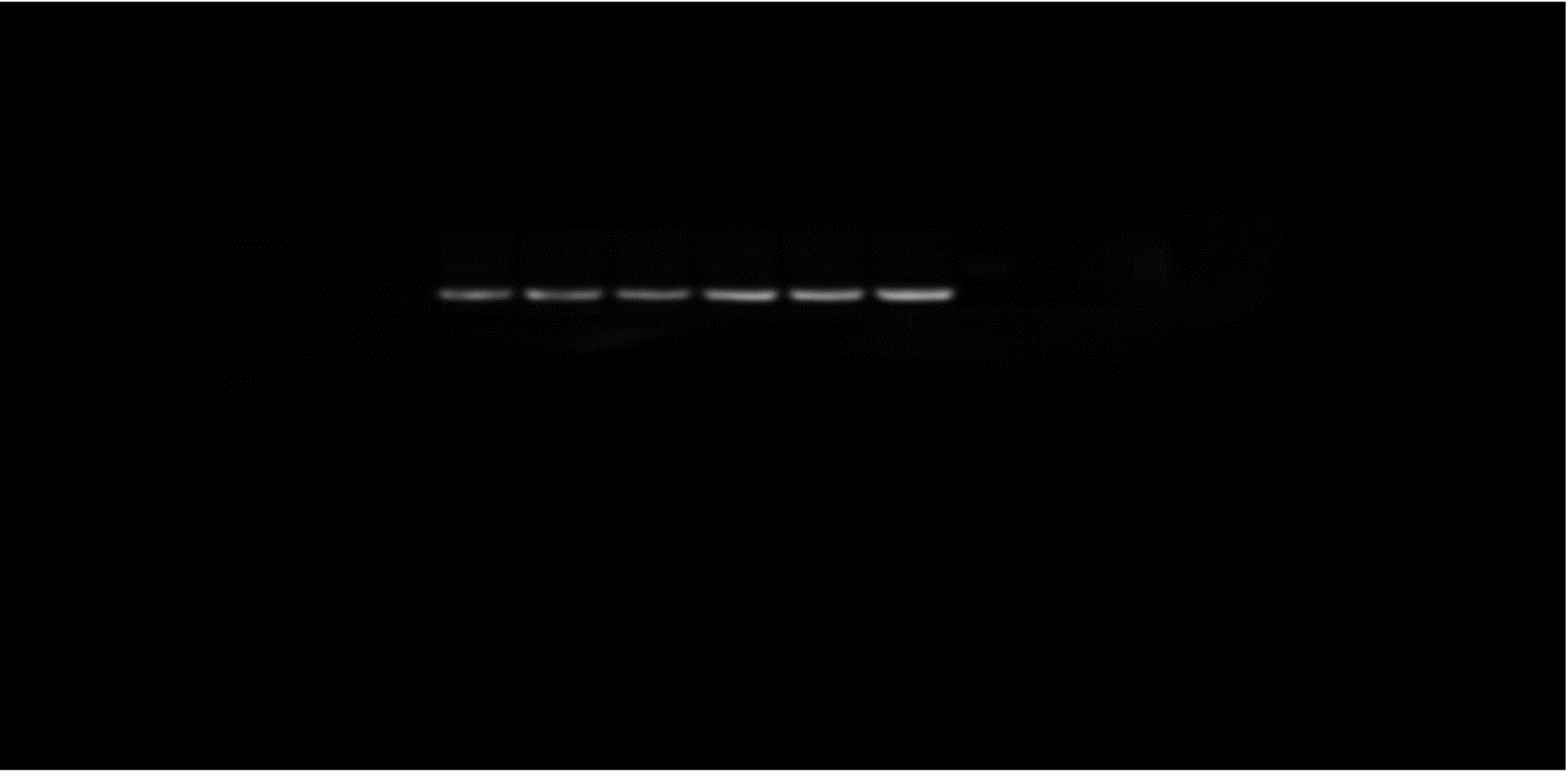

Supplement: Figure 6—source data 1. [file elife-85862-fig6-data1.zip › 6h/AsPC-1-BACTIN.tif]

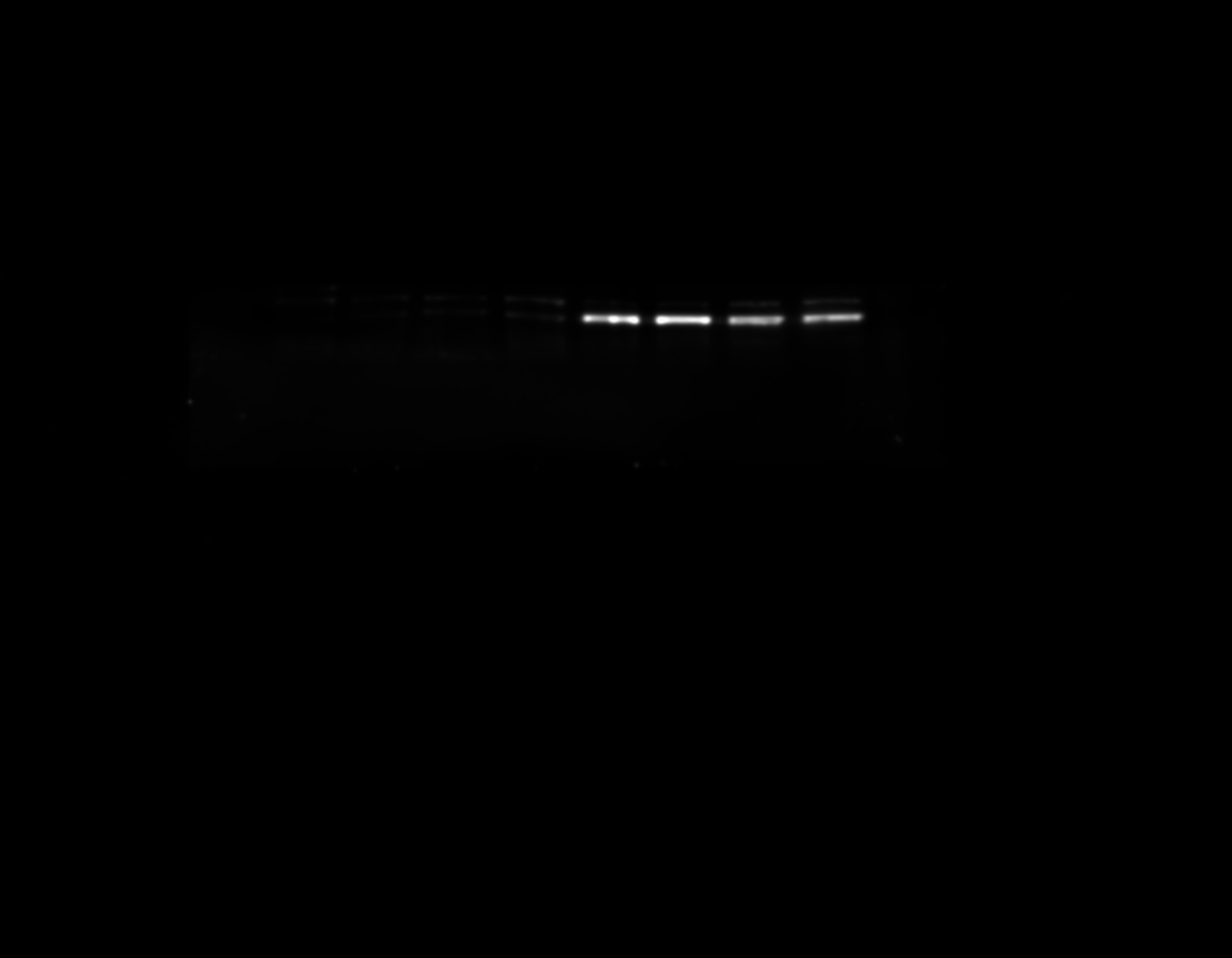

Supplement: Figure 6—source data 1. [file elife-85862-fig6-data1.zip › 6h/U049MAI-BCL2.tif]

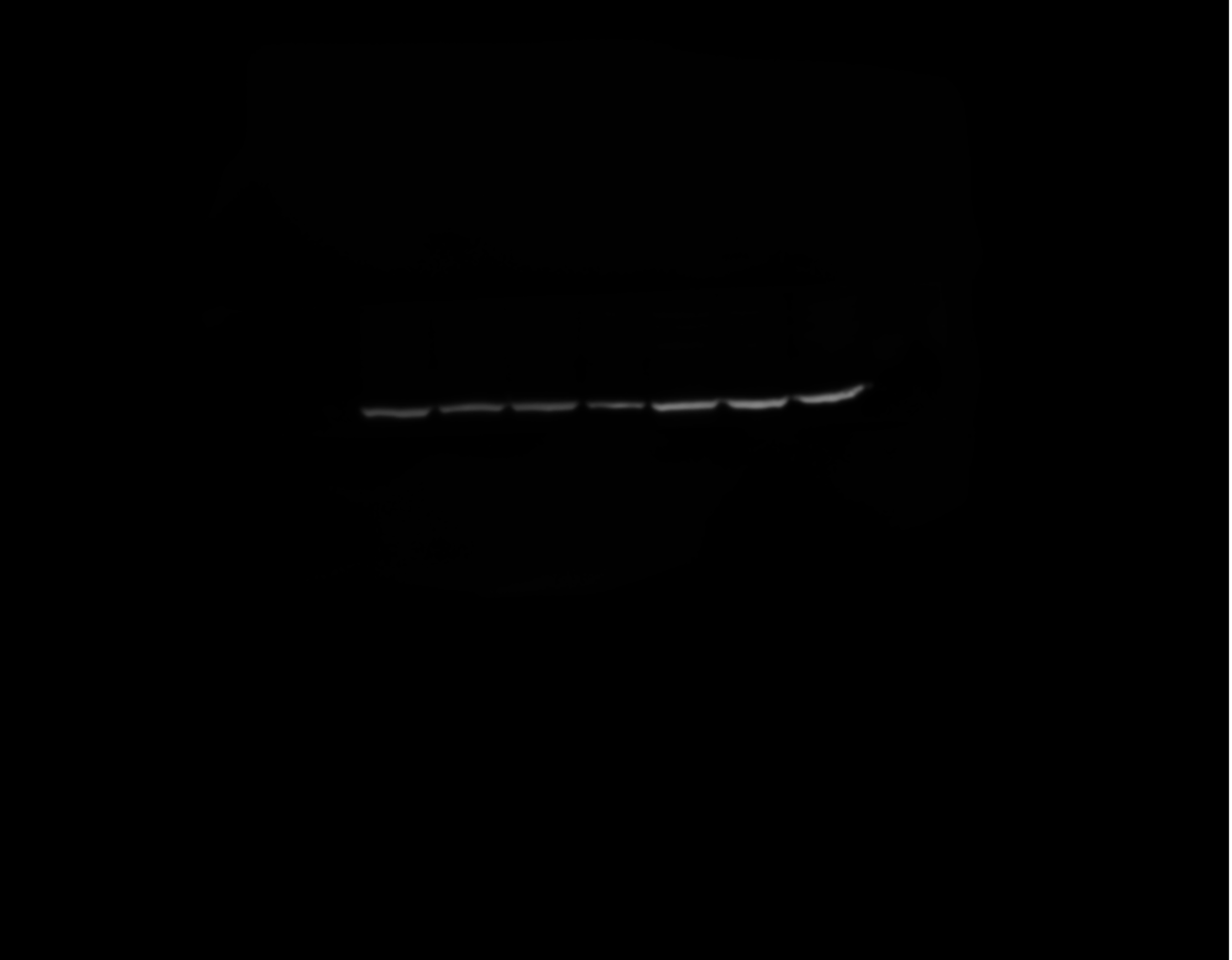

Supplement: Figure 6—source data 1. [file elife-85862-fig6-data1.zip › 6i/U049MAI-U12MIS-T-BACTIN.tif]

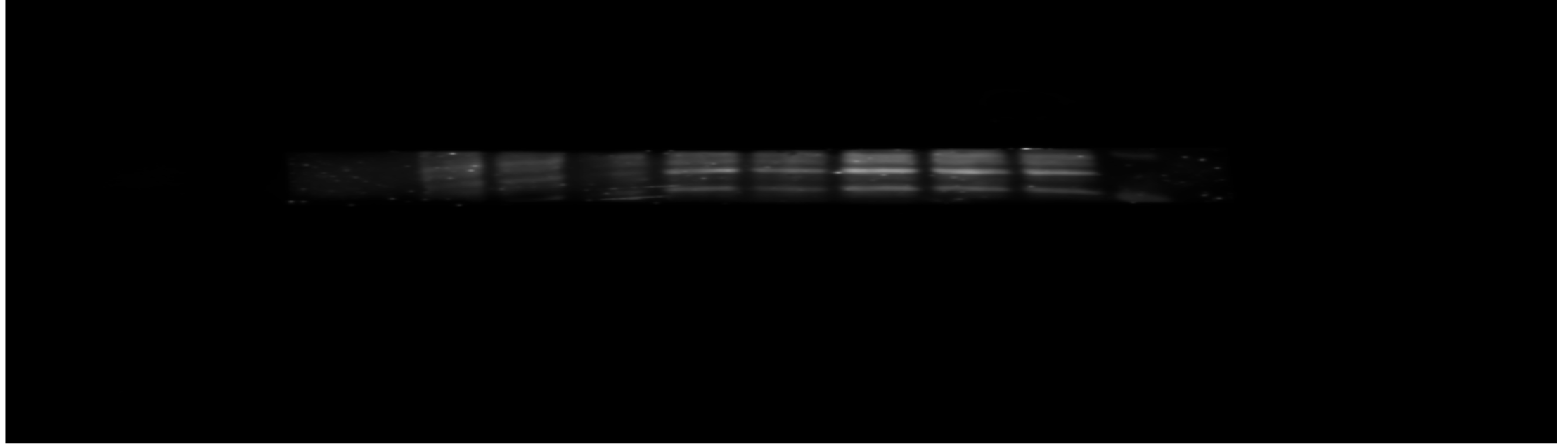

Supplement: Figure 6—source data 1. [file elife-85862-fig6-data1.zip › 6i/PANC1-CASPASE.tif]

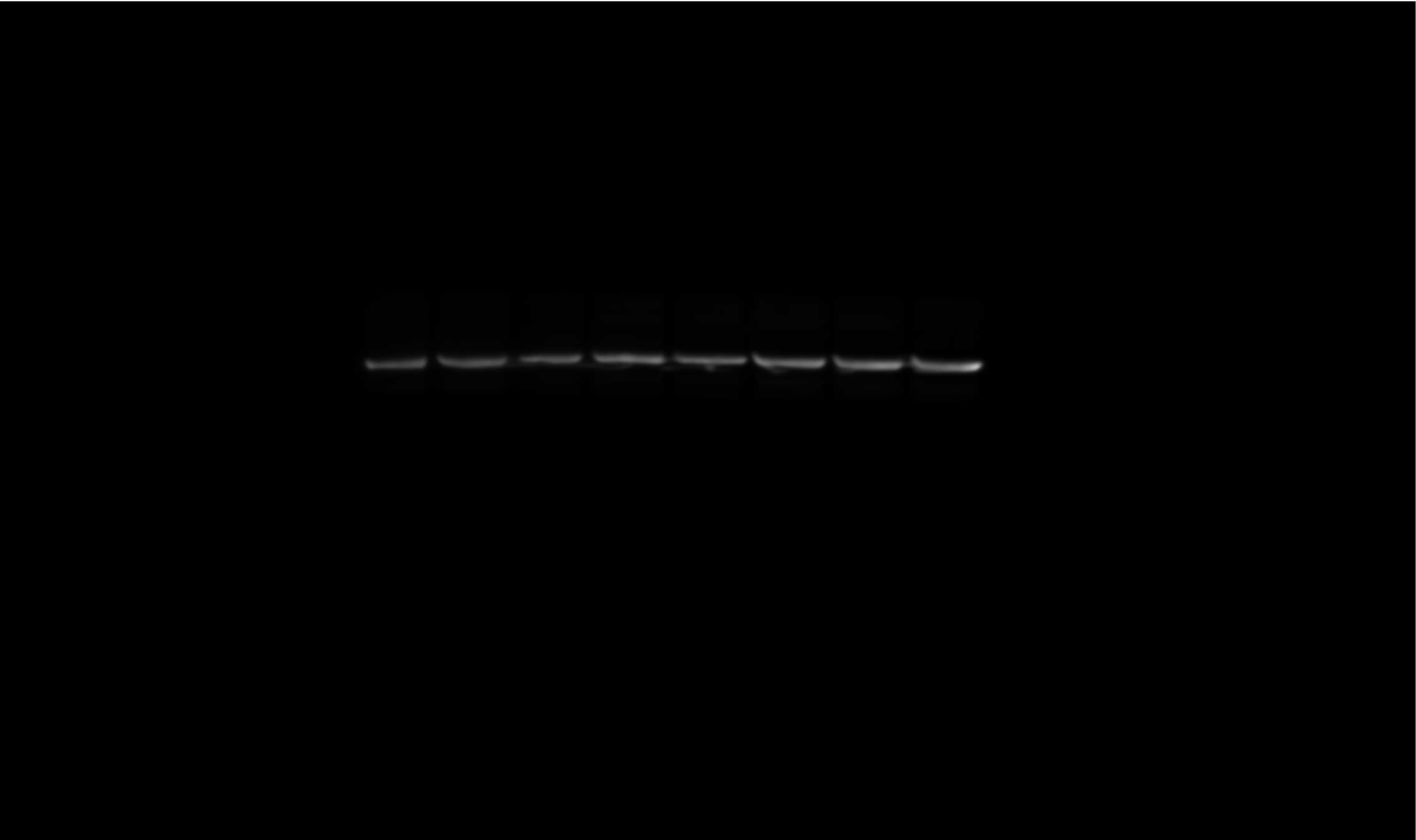

Supplement: Figure 6—source data 1. [file elife-85862-fig6-data1.zip › 6i/PANC1-BACTIN.tif]

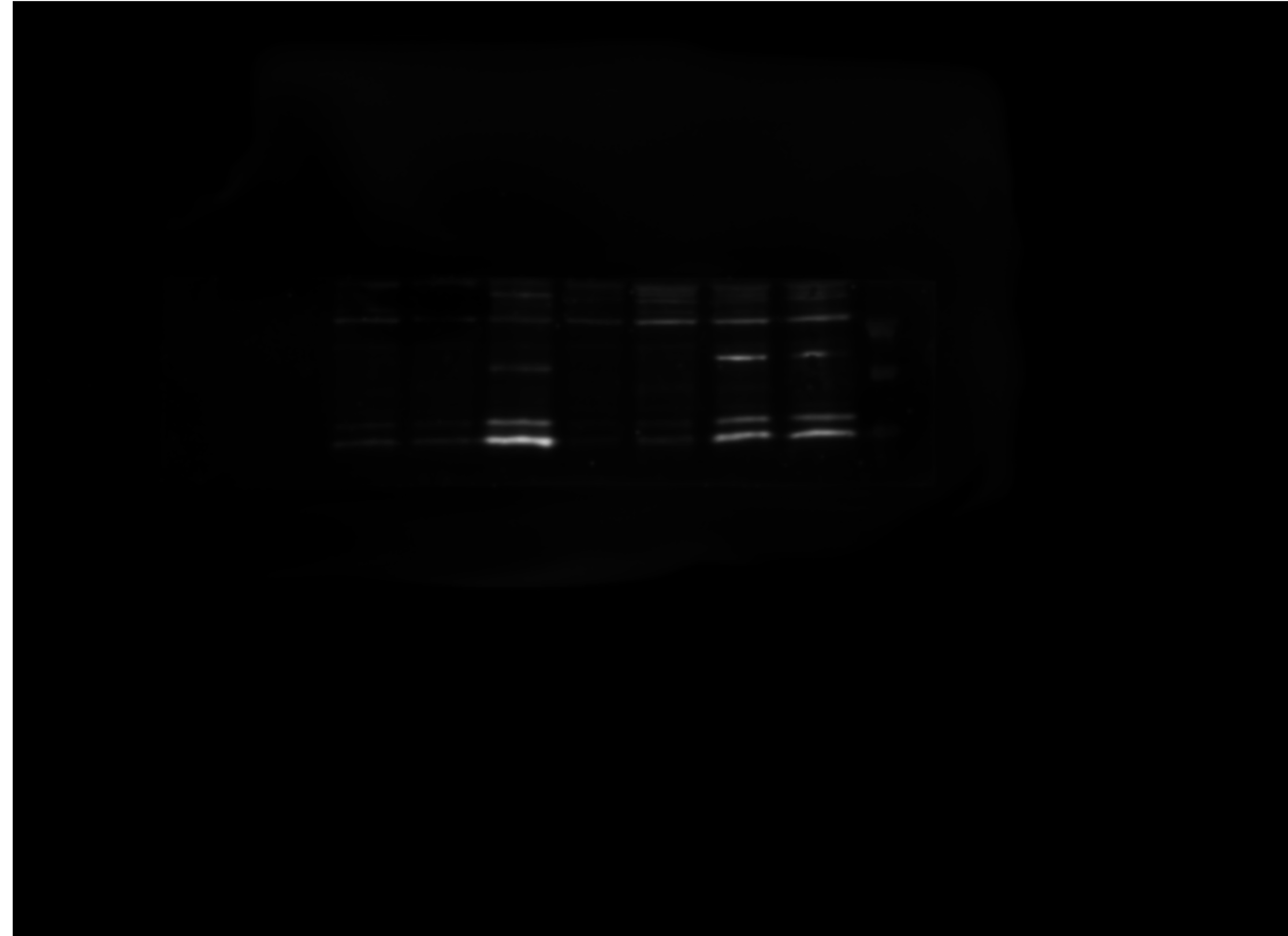

Supplement: Figure 6—source data 1. [file elife-85862-fig6-data1.zip › 6i/U049MAI-U12MIS-T-CASPASE.tif]
